# Survival, growth, and functional traits of tropical wet forest tree seedlings across an experimental soil moisture gradient in Puerto Rico

**DOI:** 10.1002/ece3.11095

**Published:** 2024-03-03

**Authors:** David Matlaga, Roel Lammerant, J. Aaron Hogan, María Uriarte, Celimar Rodriguez‐Valle, Jess K. Zimmerman, Robert Muscarella

**Affiliations:** ^1^ Department of Biology Susquehanna University Selinsgrove Pennsylvania USA; ^2^ Plant Ecology and Evolution Uppsala University Uppsala Sweden; ^3^ Tvärminne Zoological Station University of Helsinki Hanko Finland; ^4^ Department of Biology University of Florida Gainesville Florida USA; ^5^ Department of Ecology, Evolution and Environmental Biology Columbia University New York New York USA; ^6^ Department of Environmental Sciences University of Puerto Rico‐Rio Piedras San Juan Puerto Rico USA

**Keywords:** acquisitive, conservative, drought sensitivity, drought tolerance, Puerto Rico

## Abstract

Droughts are predicted to become more frequent and intense in many tropical regions, which may cause shifts in plant community composition. Especially in diverse tropical communities, understanding how traits mediate demographic responses to drought can help provide insight into the effects of climate change on these ecosystems. To understand tropical tree responses to reduced soil moisture, we grew seedlings of eight species across an experimental soil moisture gradient at the Luquillo Experimental Forest, Puerto Rico. We quantified survival and growth over an 8‐month period and characterized demographic responses in terms of tolerance to low soil moisture—defined as survival and growth rates under low soil moisture conditions—and sensitivity to variation in soil moisture—defined as more pronounced changes in demographic rates across the observed range of soil moisture. We then compared demographic responses with interspecific variation in a suite of 11 (root, stem, and leaf) functional traits, measured on individuals that survived the experiment. Lower soil moisture was associated with reduced survival and growth but traits mediated species‐specific responses. Species with relatively conservative traits (e.g., high leaf mass per area), had higher survival at low soil moisture whereas species with more extensive root systems were more sensitive to soil moisture, in that they exhibited more pronounced changes in growth across the experimental soil moisture gradient. Our results suggest that increasing drought will favor species with more conservative traits that confer greater survival in low soil moisture conditions.

## Graphical abstract

Droughts are predicted to become more frequent and intense in many tropical regions, which may cause shifts in plant community composition. We grew seedlings of eight species across an experimental gradient of soil moisture at the Luquillo Experimental Forest, Puerto Rico. Our results suggest that increasing drought will favor species with more conservative traits that confer greater survival in low soil moisture conditions.

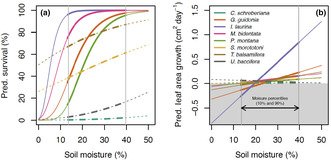

## 1 INTRODUCTION

Even under moderate scenarios of climate warming, precipitation in many tropical regions is projected to decline and become more variable in the coming decades (Feng et al., 2013; Lehner et al., 2017; Pendergrass & Hartmann, 2014). The Caribbean, for example, is expected to face a 5%–50% reduction in annual rainfall by 2100 (Herrera & Ault, 2017; Mote et al., 2017; Ramseyer et al., 2019; Taylor et al., 2018). These changes will influence vegetation through the combined impacts of warming and drying of the air and soil. Species are likely to have different responses to the impacts of climate change due, in part, to differences in their ability to resist or avoid the effects of drought (McDowell et al., 2018; Poorter & Markesteijn, 2008; Smith‐Martin et al., 2022a). As a result, forecasting the consequences of altered precipitation regimes on tropical forest ecosystems requires a solid understanding of the range of species responses to novel conditions (Comita & Engelbrecht, 2014; Meir et al., 2015).

**FIGURE 1:**
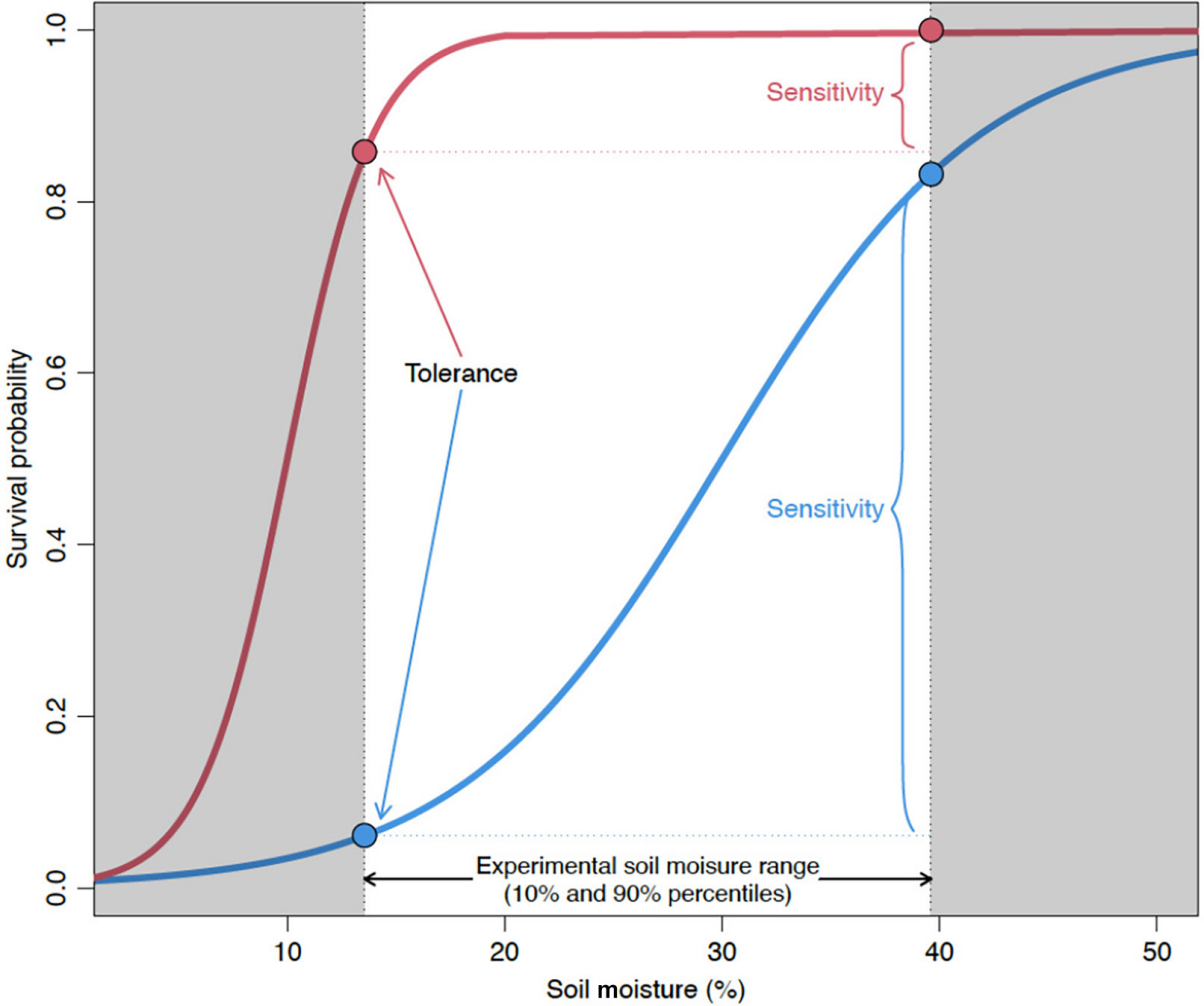
Schematic diagram illustrating metrics of survival tolerance and sensitivity to soil moisture (an analogous approach was used for growth). The two curves show the probability of survival as a function of soil moisture for two hypothetical species. We define “tolerance” as the predicted probability of survival (or growth) at the 10th percentile value of soil moisture observed in our experiment. We define “sensitivity” as the difference in the predicted probability of survival (or growth) between the 90th and 10th percentile values of soil moisture observed in our experiment. In the diagram, the red curve represents a species with relatively high tolerance and low sensitivity to low soil moisture. The blue curve, in contrast, represents a species with a relatively low tolerance and high sensitivity to low soil moisture. In our dataset, the 10th and 90th percentile values of soil moisture correspond to 13.5% and 39.1%, respectively.

One challenge for understanding how different species will respond to drought is the variety of ways to characterize demographic responses (Figure 1). For example, species may differ in their ability to survive or grow under extremely low resource conditions (e.g., periods of very dry soil; Tilman, 1982). Species could also differ in the degree to which their demographic rates are sensitive to variation in environmental conditions. More specifically, the relative magnitude by which a given demographic rate varies across a relevant range of environmental stress can be used to characterize species' responses (Engelbrecht et al., 2007; Rüger et al., 2020). Additionally, different vital rates (e.g., growth and survival) may exhibit different responses to drought (Yang et al., 2022), making it critical to evaluate multiple vital rates for a more complete picture of responses to environmental variation. Correlations among responses for different vital rates could reflect trade‐offs that underlie demographic variation in diverse species groups (e.g., Russo et al., 2021). Overall, a solid understanding of species responses to environmental change requires a comprehensive evaluation of responses for multiple demographic rates.

Diverse responses of tropical trees to drought should be linked, at least in part, to traits that reflect their resource strategies (O'Brien et al., 2017; Oliveira et al., 2021; Paz et al., 2015; Smith‐Martin et al., 2022a). For example, species with relatively “acquisitive” traits (e.g., lower wood density [WD], leaf mass per area [LMA], and root tissue density [RTD]) tend to maximize growth rates by producing “cheap” tissues (i.e., low carbon investment) with efficient hydraulic architectures (Castro‐Díez et al., 1998; Gilbert et al., 2006; van Gelder et al., 2006; Markesteijn, Poorter, Bongers, et al., 2011; Markesteijn, Poorter, Paz, et al., 2011). While these characteristics may enable a rapid demographic response to changes in resource availability (i.e., greater sensitivity), efficient hydraulic systems also tend to be more susceptible to drought‐induced mortality (i.e., less tolerant) (Castro‐Díez et al., 1998; van Gelder et al., 2006; Mantova et al., 2021; Niinemets & Valladares, 2006; Smith‐Martin et al., 2022a). In contrast, species with relatively “conservative” functional strategies (e.g., higher WD, LMA, and RTD) tend to exhibit lower maximum growth rates but greater survival during low resource conditions, including drought (Gilbert et al., 2006; Markesteijn, Poorter, Bongers, et al., 2011; Markesteijn, Poorter, Paz, et al., 2011). In other words, species with more conservative traits may be better able to tolerate dry conditions (drought resistance) whereas species with more acquisitive traits may be more sensitive (or responsive) to shifts in water availability (Bartlett et al., 2019; Duque et al., 2015; Esquivel‐Muelbert et al., 2018). Nevertheless, the role of traits in modifying various aspects of tree demographic responses to drought in tropical humid forests remains understudied and thus limits our ability to forecast the consequences of altered precipitation regimes on tropical forest ecosystems.

The selective pressure exerted by altered precipitation regimes on community composition may be particularly strong for early life stages of trees. The seedling life stage, in particular, represents a major recruitment bottleneck and seedling survival rates can influence future tree community composition (Grime, 1979; Poorter, 2007). Additionally, because of their shallow root systems, seedlings can be particularly strongly affected by drought (Comita & Engelbrecht, 2014; Harrison & LaForgia, 2019; Uriarte et al., 2018; Wright & Westoby, 1999). Seedling traits related to drought response may, therefore, play a particularly important role in determining how drought conditions impact the future composition of tropical forests.

To address these issues, we evaluated responses of wet tropical forest seedlings to variation in soil moisture using an 8‐month seedling drought‐exposure experiment, where eight common tree species representing a broad range of life‐history strategies were subjected to an experimental gradient of soil moisture via rainfall manipulation. We monitored seedling growth and survival for the duration of the experiment and then used linear regression to relate metrics of demographic performance with a suite of functional traits to better understand how traits mediate demographic response to drought conditions. We addressed the following questions:

*How do species vary in their demographic performance across an experimental soil moisture gradient? And, is variation in demographic rates related to species functional traits?* We expected species to vary considerably with respect to survival and growth responses along the soil moisture gradient based on their different life‐history strategies. We expected species with relatively conservative traits (e.g., low LMA, WD, and RTD) to have higher overall survival and lower overall growth compared to species with more acquisitive traits. Along the soil moisture gradient, we expect species with relatively conservative traits to exhibit higher tolerance to drought (i.e., higher rates of survival and growth under low soil moisture conditions) compared to species with relatively acquisitive traits (Table S1). At the same time, we expected species with relatively acquisitive traits to exhibit higher sensitivity to soil moisture variation (i.e., more pronounced changes in demographic rates) compared to species with more conservative traits (Hiromi et al., 2012; Markesteijn, Poorter, Bongers, et al., 2011; Markesteijn, Poorter, Paz, et al., 2011; Poorter & Markesteijn, 2008).

## 2 METHODS

### 2.1 Study site

The experiment was conducted at the El Verde Research Area, within the Luquillo Experimental Forest (LEF), Puerto Rico (18°32′ N, 65°81′ W). The LEF is classified as a subtropical wet montane forest with a mean annual rainfall of 3500 mm (±725 SD) and a mean temperature of 23°C (Daly et al., 2003; Ewel & Whitmore, 1973; Thompson et al., 2002). Rainfall is aseasonal, with no month typically receiving <200 mm. Nevertheless, half of the annual precipitation occurs between August and December, with considerably drier conditions between January and March. Rainless periods of ~1 week are common (once per year, on average) while longer droughts (>2 weeks) have historically occurred every few years (Scatena et al., 2012). Long‐term data show declining rainfall in the LEF (Méndez‐Lazaro et al., 2023; Moraes et al., 2022), a trend that is supported by modeling results (Ramseyer et al., 2019). The dominant soil types in the LEF are Zarzal, a deep and well‐drained oxisol, and Cristal, a deep but poorly drained ultisol (Mount & Lynn, 2004). The area comprises both primary and secondary forests that have been free from human disturbance since the 1940s (Thompson et al., 2002).

### 2.2 Experimental set‐up and plot of physical conditions

We collected seeds of eight woody species (seven trees and one palm; Table S2) that were fruiting between December 2018 and March 2019. These species represent a range of life‐history strategies and account for about 44% of the total individuals >1 cm DBH in the nearby 16‐ha Luquillo Forest Dynamics Plot (Thompson et al., 2002). Seeds were cleaned, spread on soil in horticultural trays, and then covered with a thin layer of soil. The soil we used was obtained from within 100 m of the field station, which had similar properties to the soil at the experimental sites (JK Zimmerman, *personal observations*). Trays were placed under shade cloth (approximately 60%–70% shading) and watered daily until seedling emergence. Within a week of emergence, individuals were transplanted to pots (ca. 10 cm diameter, 12 cm deep) and placed under shade cloth.

We set up our experiment along the top of two ridges within 1 km of El Verde Field Station. Ridge tops were selected to minimize water flow into the plots and to maximize drought stress (O'Connell et al., 2018). In May 2019, we established 60 seedling plots (30 drought and 30 control, randomly assigned), where 16 seedlings (2 per species) were transplanted randomly in a 4 × 4 grid with 10 cm between each seedling (Figure S1). Individual seedlings were randomly assigned to plots and locations within the grid. After a 3‐week acclimation period, we constructed rain‐out shelters directly over each grid of seedlings using PVC frames with a 1.2 m × 1.2 m SUNTUF® corrugated polycarbonate roof (Palram Industries Ltd. Kutztown, PA, USA) and a PVC gutter on the downslope side (Figure S1). According to the manufacturer's specifications, the roofing material reduces light transmission by 10% (https://www.palram.com/us/product/suntuf‐diy‐polycarbonate‐corrugated‐sheets/). For drought plots, precipitation from the gutter ran into a hose directed away from the plot. For control plots, precipitation runoff from the roof was collected in a 5‐gallon bucket and poured back onto the seedlings using a watering can within ~24 h of each rain event (greater than 5 mm precipitation over 24 h) during the experiment. We dug trenches on the upslope edge of droughted plots and buried a small (~13 cm wide) strip of polycarbonate corrugated roof panel approximately 10 cm deep, leaving a 3 cm lip above ground to prevent downslope water flow. A sham trenching was performed for control shelters. We scrubbed roofs with a soft brush as needed to prevent algae and lichen growth which could reduce light.

In each plot, we measured volumetric soil water content at 0–12 cm (hereafter soil moisture) depth every 2 weeks using a handheld probe (Campbell Scientific HS2 system, Queensland, Australia). Four measurements were taken at the corners of the seedling grid. Because measured values of soil moisture in plots spanned a continuum, we treated soil moisture as a continuous variable in analyses rather than a binary treatment effect (although we summarize treatment effects on soil moisture in Figure S2). To validate that the soil moisture conditions reflect a gradient of drought stress from the plant perspective, we used a stable carbon isotope analysis of leaf tissues (Cernusak et al., 2013) to assess the degree of integrated drought stress experienced by seedlings in our experiment (Table S3).

We measured canopy openness for each plot using a spherical densiometer (Forestry Suppliers, Jackson, MS) at the end of the experiment. We averaged measurements from two observers who independently recorded densiometer measurements above the center of each shelter. Note that our study area was impacted by a major hurricane (María) in September 2017 (Leitold et al., 2021) and canopy openness levels during our study were still somewhat higher than typical for closed‐canopy conditions. To determine if shelters altered the temperature experienced by seedlings, we placed temperature loggers (DS1921G Thermochron iButton, Maxim/Dallas Semiconductor Corp., USA) in plastic bags and hung them 5 cm from the soil surface in the center of 8 randomly chosen shelters as well as 2 m from the shelters. Loggers recorded temperature hourly over 3 days (July 31, 2020, to August 2, 2020). We compared average hourly temperature between sheltered and adjacent un‐sheltered areas using a paired *t*‐test.

### 2.3 Seedling demography and functional traits

For each seedling, we recorded total initial leaf area (cm^2^), monitored survival weekly, and measured the length of all leaves monthly. We then estimated total leaf area (and leaf area growth) using species‐specific leaf length‐leaf area relationships from data collected on shade house grown seedlings (Table S3).

At the end of the experiment, all surviving seedlings were harvested to measure a suite of functional traits. Seedlings were excavated, keeping their root systems intact, and rinsed with water. We immediately measured maximum root length, basal diameter (cm), and leaf thickness (mm). We then sectioned seedlings by organ (i.e., leaves, and roots), which were weighed for fresh mass and then scanned (CanoScan Lide 400) to estimate leaf area (cm^2^) and several root traits (see below). All parts were dried at 70°C until constant mass (for ≥72 h) before recording dry mass. Total area of fresh leaves was determined with ImageJ (Schneider et al., 2012). Root system scans were edited by hand to improve their contrast and then we used WinRHIZO (2012 version, Regent Instrument, Quebec, Canada) to measure root system morphology. In total, we quantified the following 11 traits for each surviving seedling: leaf area (cm^2^), leaf mass per area (LMA; kg/m^2^), leaf thickness (mm), leaf dry matter content (%), root:shoot mass ratio (%), specific root length (SRL, cm/g), root tissue density (RTD, g/cm^3^), total root system length (cm), average root system diameter (mm), maximum rooting depth (cm), and number of root tips. We log‐transformed all traits (except LDMC) comply with normality assumptions prior to analyses.

Several traits were strongly correlated (Table S4) and therefore we used Principal Component Analysis (PCA) to reduce the dimensionality of the trait data and explore the extent to which multivariate phenotypes were associated with demographic responses to the soil moisture gradient. Using log‐transformed variables, we first fit a PCA using the “prcomp” function in R v 4.2.0 (R Development Core Team, 2020). Then, to clarify the relationships among the included traits, we performed a varimax rotation on the first and second PCA axes. In subsequent analyses, we used values along the rotated component (RC) axes to reflect multivariate phenotypes in addition to the univariate trait values.

To directly assess the effect of the experimental treatments on seedling physiological performance, we measured leaf intrinsic water‐use efficiency (iWUE) using carbon (C) isotopes. Isotopically‐derived iWUE is an indicator of plant C assimilation and *g*_sw_ (stomatal conductance to water vapor slope) at intermediate time scales (weeks to months in the context of this experiment). It integrates drought stress through isotopically derived estimates of intracellular CO_2_ (*c*_i_), which decreases as *g*_sw_ decreases with increasing drought stress (Cernusak et al., 2013; Farquhar et al., 1982). Leaf tissue elemental analyses of C, nitrogen (N), and their isotopes (^13^C and ^15^N) were completed using 10 individuals from six species (excluding *U. baccifera* and *C. shreberiana* because of low survival) from the wettest and driest plots of the experimental soil moisture gradient. Dried leaf tissues were homogenized in individual 15 mL sterile plastic vials using sterile stainless‐steel beads and a Mini‐G 1600 tissue homogenizer (SPEX Sample Prep, Metuchen, NJ). Samples were analyzed using a Carlo Erba NA 1500 Elemental Analyzer (Thermo Scientific, Waltham, MA USA), fitted with a Costech zero‐blank autosampler at the Duke Environmental Stable Isotope Laboratory (Durham, North Carolina).

Carbon isotope values were converted to intrinsic (i.e., long‐term) concentration of intracellular CO_2_ (*c*_i_) estimates using the following equation for C3 plants and a *c*_a_ value of 408 μmols/mol CO_2_: Δ = (4.4 + 2.26*c*_i_/*c*_a_) (Farquhar et al., 1982; Lambers et al., 2008). Intrinsic water use efficiency (iWUE) was determined from calculated intrinsic *c*_i_ values using the following equation, from (Lambers et al., 2008): iWUE = (*c*_a_ × (1 − *c*_i_/*c*_a_))/1.6. We used linear regressions to evaluate how seedling iWUE varied with soil moisture, fitting separate regressions for each of the six species for which were measured iWUE, and report the overall correlation between iWUE and soil moisture across all individuals measured.

### 2.4 Data analysis

We used separate modeling approaches to characterize species‐specific variation in survival and growth across the experimental soil moisture gradient. For survival, we fit species‐specific mixed effects Cox proportional‐hazards models using the R‐packages “survival” and “coxme” (Therneau, 2020a, 2020b). Specifically, the “surv” function was used to create a survival object, which contained the survival census data and was used as the response variable in a mixed linear model. We then fit a mixed effects Cox model using the “coxme” function for survival of seedling *i* in plot *p* over census interval *t* using the form:
(1)

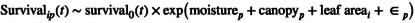

where survival_0_ signifies the seedlings status (alive or dead) at the time, *t*, moisture is the grand mean of soil moisture in plot, *p*, canopy is the plot‐level canopy openness (densiometer) measurement, leaf area is the total leaf area of the individual seedling when the experiment began (used as a predictor to account for variation in initial seedling size), and ∈_*p*_ is the plot random effect.

For growth, we used the R‐package “lme4” to fit linear mixed models (Bates et al., 2015) with daily leaf area growth rate (cm^2^/day) of individual seedlings between each census interval as the response variable. Daily growth of individual *i* in plot *p* takes the form:
(2)

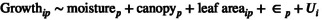

where moisture, canopy, and leaf area are as described above, ∈_*p*_ is a random effect of plot, and *U*_*i*_ is an individual random effect to account for repeated measurements. For both survival and growth models, we considered an interaction term between soil moisture and canopy openness but this did not improve model fit so we excluded it from the final models.

Based on the fitted models, we define two complementary metrics which quantitatively describe variation in two vital rates (survival and growth) across the experimental gradient of soil moisture. First, we define “sensitivity” to soil moisture variation as the change in a predicted demographic rate (survival or growth) across the relevant range of soil moisture in our experiment. Specifically, we calculated the difference between predicted survival and growth at the 10 and 90 percentile values of observed soil moisture from our plots (corresponding to 13.5% and 39.1% soil moisture, respectively) for each species. Based on this calculation, more sensitive species display a greater change in a given vital rate per unit change in soil moisture across the range of our experimental gradient. Second, we define “tolerance” to low soil moisture as the predicted vital rate (survival or growth) at the lower 10 percentile value of observed soil moisture (i.e., 13.5%, while holding other covariates at their mean values). To estimate standard errors for growth metrics, we used bootstrapping implemented with the “bootMer” function in the “lme4” R package (Bates et al., 2015). For survival metrics, we extracted standard errors of predicted values directly with the “predict” function of the “survival” R package (Therneau, 2020a).

To characterize relationships between metrics of demographic performance, within and across vital rates, we used linear regression to evaluate the relationship between sensitivity of survival and growth, tolerance of survival and growth, and between sensitivity and tolerance, for both survival and growth. To incorporate uncertainty in these comparisons, we implemented a measurement error model in the “brms” R package (Bürkner, 2017), as specified by Nicenboim (2023), that included standard errors of the demographic metrics (as described above). We used default settings and priors for model fitting and assessed model convergence using the Rhat statistic and visual inspection of trace plots.

To evaluate the relationship between seedling traits and demographic performance, we used linear measurement error regression models (as above) to relate species multivariate trait axes (RC1 and RC2) with the sensitivity and tolerance metrics for both survival and growth. We included standard errors of the demographic metrics (as above) as well as standard errors for the positions along trait axes RC1 and RC2 for each species. All analyses were conducted in R v 4.2.0 (R Development Core Team, 2020).

## 3 RESULTS

Across the plots, average soil moisture calculated over the duration of the experiment ranged from 9% to 49% (median 27.8 ± SD 8.4) (Figure S2). The rank order of plots in terms of soil moisture was consistent and highly correlated with the minimum soil moisture per plot (Pearson's *r* = .7, *p* < .01). Canopy openness values ranged from 2% to 20% (median 9.1 ± SD 3.5) and these values were not significantly correlated with soil moisture (Pearson's *r* = −.17, *p* = .18). There was no significant difference in average hourly temperature measured in sheltered and adjacent un‐sheltered areas (*t* = −1.25, *df* = 14, *p* = .23). Four of six species tested (except *P. montana* and *T. balsamifera*) showed significant negative correlations between intrinsic water‐use efficiency (iWUE) and soil moisture (Figure S3; Table S4), suggesting that lower soil moisture led to more integrated drought stress during the experiment (across all samples Pearson's *r* = −.28, *p =* .002).

### 3.1 Seedling demographic responses

**FIGURE 2:**
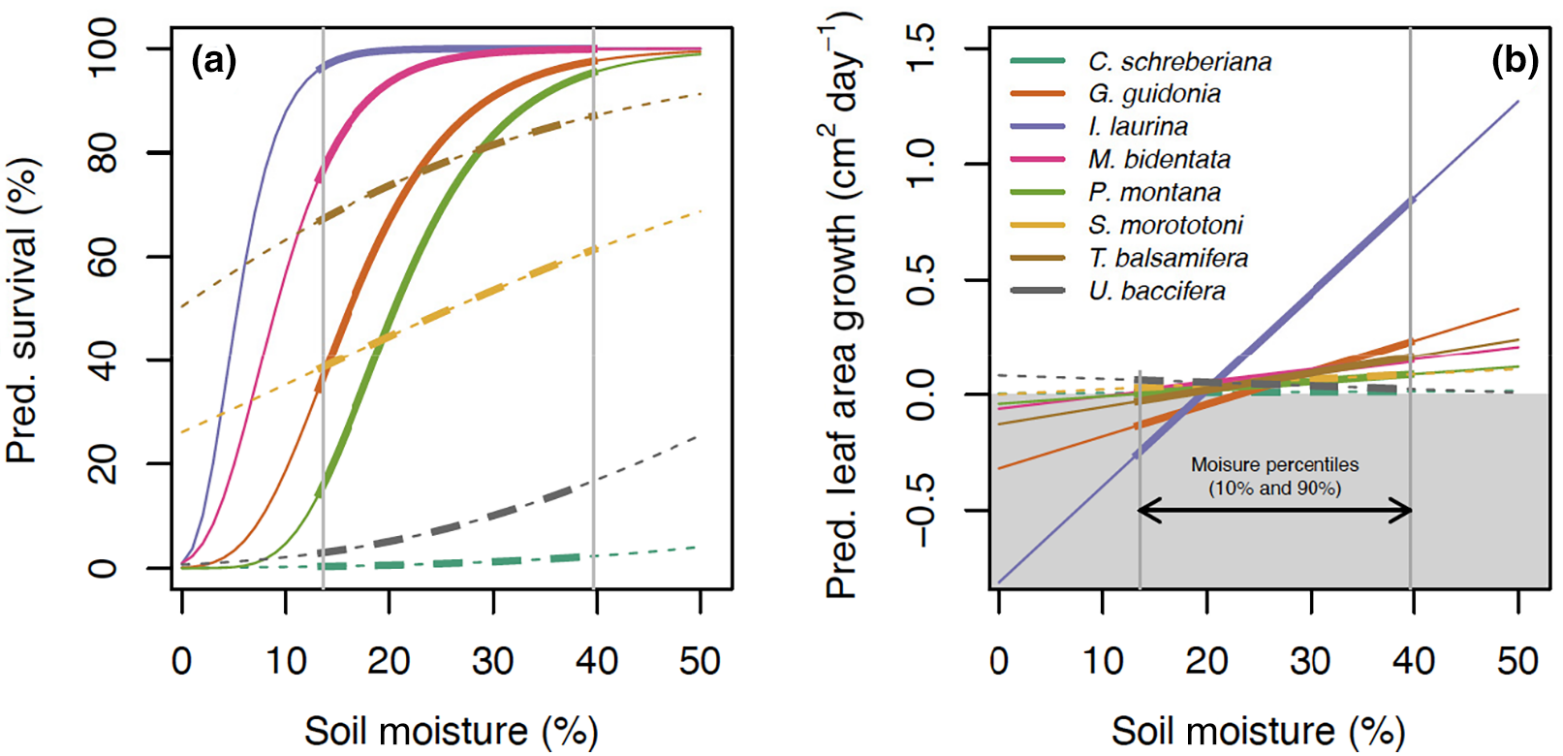
(a) Predicted model fits for survival for eight seedling species across the experimentally induced soil moisture gradient. Solid lines show model fits where soil moisture had a statistically significant effect on survival, whereas dashed lines show fits with non‐significant soil‐moisture effects. The point where lines cross the left‐most vertical line represents “tolerance”, that is, survival at the 10th percentile of soil moisture measurements; “sensitivity” is based on the difference in predicted survival at the 90th and 10th percentiles of soil moisture observations. (b) Predicted model fits for leaf area growth in relation to soil moisture. The gray area indicates negative leaf area growth rates (leaf area decrease) by species. Solid lines represent statistically significant slopes.

Species varied considerably in their survival; four had >80% overall survival (*G. guidonia*, *I. laurina*, *T. balsamifera*, and *M. bidentata*), while two had <20% overall survival (*C. schreberiana* and *U. baccifera*) (Figure 2a; Figure S4). Survival generally increased with soil moisture, but the effect varied substantially among species with significant positive effects for half the species. Survival was negatively associated with canopy openness for four species, positively associated with starting size for one species (*Urera baccifera*), and negatively associated with starting size for two species (*Guarea guidonia* and *Prestoea acuminata* var. *montana*) (Table S7).

Species also varied substantially in their growth rates along the soil moisture gradient (Figure 2b; Figure S5). For example, among seedlings that survived until the end of the experiment, total leaf area growth ranged from −1.32 to 4.16 mm^2^/day (−3.02 to 9.53 cm^2^ total), with negative leaf area growth indicating leaf loss through herbivory or senescence possibly due to water stress. As with survival, leaf area growth rates were generally positively associated with soil moisture, although the effect was only significant for six of the eight species. Most species were predicted to have negative leaf area growth rates in plots with soil moisture less than ~20%. Leaf area growth was positively associated with canopy openness for two species and negatively associated with starting size for all species (Table S8).

Average growth and survival rates were not significantly correlated (Table S9). Additionally, we did not find strong evidence for correlations between the sensitivity and tolerance metrics within or across vital rates (Table S9). Specifically, there were no significant relationships between sensitivity of survival and growth, tolerance of survival and growth, or between survival sensitivity and tolerance. Growth sensitivity was significantly negatively related with growth tolerance, which corresponds to an expected link between the slope and intercept terms in linear models (i.e., more sensitive species [those with steeper slopes] had greater tolerance [lower intercepts]).

### 3.2 Trait‐mediated demographic responses

**FIGURE 3:**
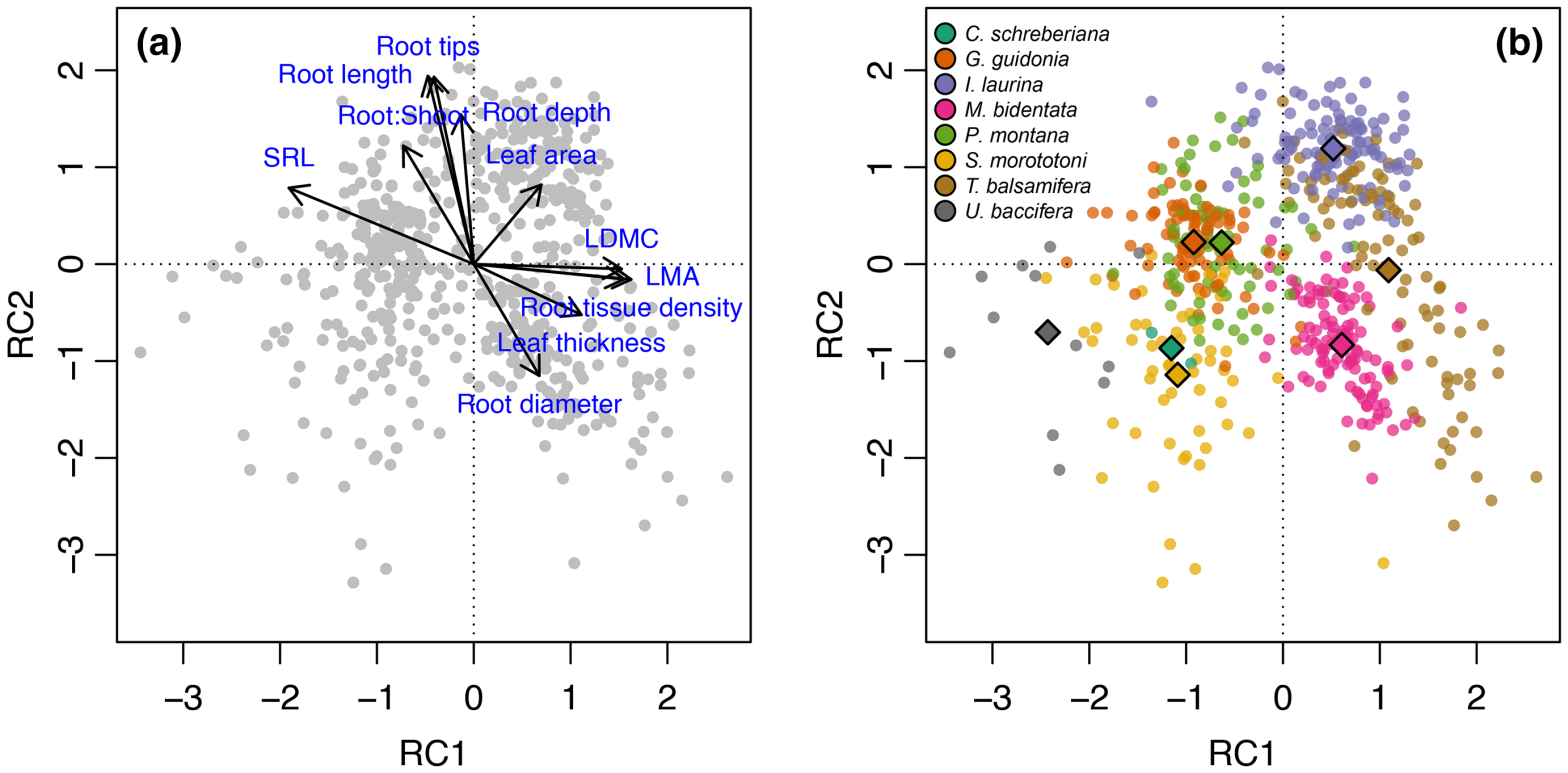
Varimax‐rotated Principal Component Analysis of functional traits for all surviving seedlings. (a) Variable ordination biplot, showing the functional trait space condensed to two ordination axes. Gray points show positions of individual seedlings. (b) Position of seedlings within two‐dimensional functional trait space; individuals colored by species. Diamond points show mean position in ordination space across individuals of each species.

Functional traits varied extensively both among and within species. The varimax‐rotated PCA revealed a separation among species that was broadly consistent with an acquisitive‐to‐conservative spectrum of multivariate phenotypes (Figure 3; Table S6). Rotated component 1 (RC1) explained 32% of the total trait variation and primarily represented an axis of carbon investment in leaf and root tissues (i.e., higher values of LDMC, LMA, RTD, and lower values of SRL). Rotated component 2 (RC2) explained an additional 32% of the variation and reflected increasing investment in belowground tissue (i.e., larger total root system length, more root tips, and a higher root‐to‐shoot ratio).

Species average survival was positively associated with RC axis 1 (estimated slope [95% credible interval (CI)]: 25.26 [8.53–43.86]), as well as RTD (93.21 [12.08–174.73]), suggesting that species with more conservative trait values (greater resource investment in leaf and root tissue) tended to have higher overall survival (Table S10). Average growth rates were positively associated with total rooting depth (3.12 [0.16–5.67]), reflecting higher overall growth rates for species with more extensive root systems (Table S10).

**FIGURE 4:**
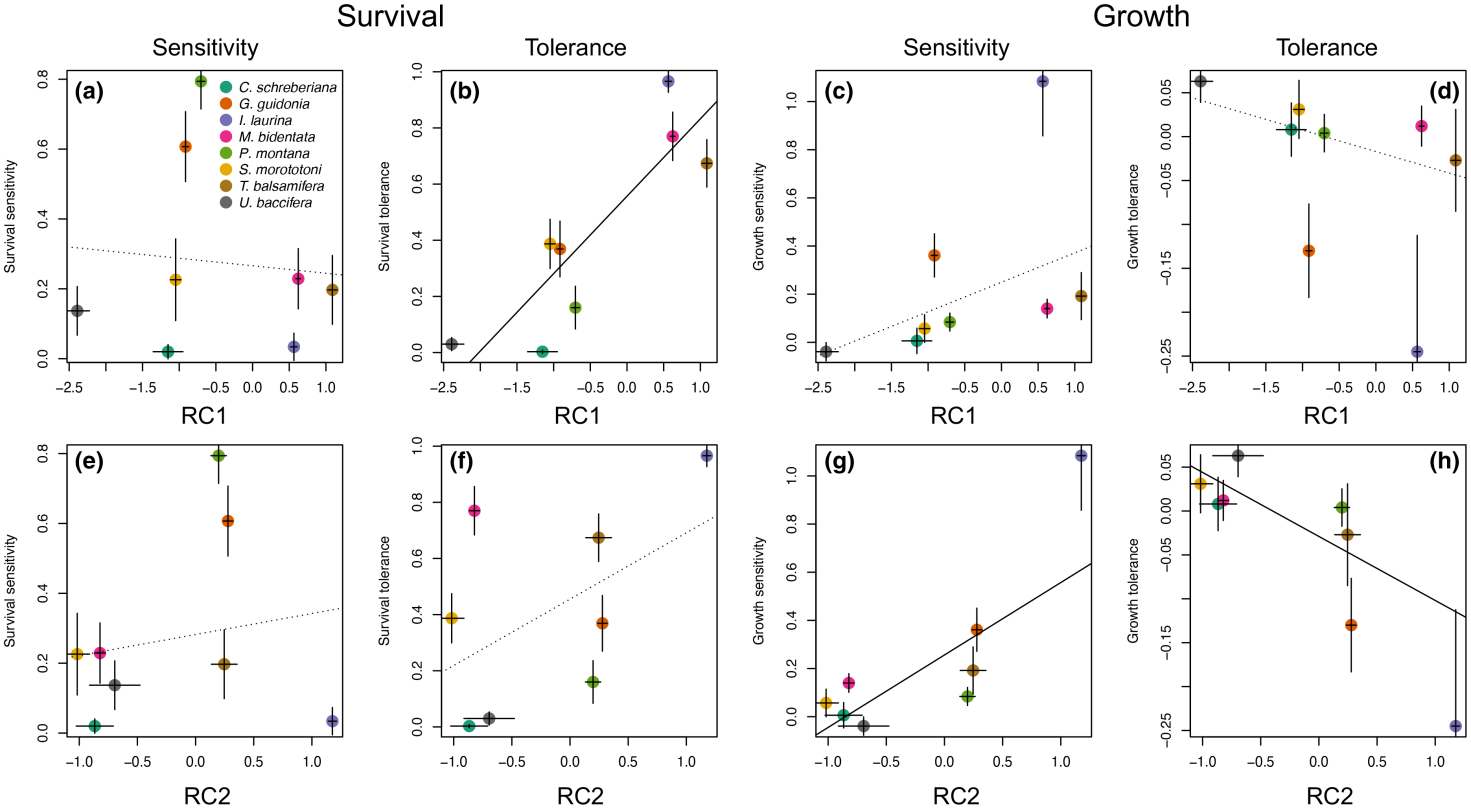
Bivariate associations between demographic metrics (tolerance and sensitivity of survival and growth) and the first two RC axes of the trait ordination. See main text and Figure 1 legend for definitions of the tolerance and sensitivity metrics. Error bars show one standard error on estimates of demographic metrics and trait ordination values. Trend lines are based on fitted Bayesian models that include measurement error (see Section 2); lines are solid when 95% credible intervals of slope estimates do not overlap zero.

We found several significant associations between traits and the metrics of sensitivity and tolerance (Figure 4). Here we highlight results based on multivariate trait axes and we provide full results for univariate traits in Table S10. Sensitivity of survival to soil moisture (i.e., difference in predicted survival at the 90 and 10 percentile values of observed soil moisture) was not significantly related with either of the RC axes nor any of the individual traits. Sensitivity of growth was, however, positively associated with RC2 (estimated slope [95% CI]: 0.30 [0.07–0.60]) and three individual root traits (total length, depth, and number of tips) (Table S10), which together reflect more pronounced growth responses for species with larger root systems.

Tolerance of survival to low soil moisture (i.e., survival predicted at the lower 10 percentile of observed soil moisture) was positively related to RC1 (estimated slope [95% CI]: 0.25 [0.12–0.54]), as well as two leaf traits (LMA and LDMC) and one root trait (RTD), overall suggesting that species with relatively conservative traits had higher survival under low soil moisture conditions. Growth tolerance to low soil moisture was negatively related to RC2 (−0.07 [−0.16 to −0.001]), R:S ratio, and two individual root traits (total length and total depth), indicating that species with relatively smaller root systems had higher growth rates in low soil moisture conditions.

## 4 DISCUSSION

We used a seedling drought experiment in an aseasonal subtropical forest to relate demographic responses along a soil moisture gradient with a suite of above‐ and below‐ground traits. Our key findings show that species‐specific survival and growth responses were related to traits such that: (1) species with more conservative functional traits had overall higher average survival (irrespective of soil moisture), as well as higher survival in low soil moisture conditions (i.e., higher tolerance) compared to species with more acquisitive traits; (2) species with more extensive root systems had greater leaf area growth, more pronounced responses of growth to the soil moisture gradient (i.e., higher growth sensitivity), and lower growth in low soil moisture conditions (i.e., lower growth tolerance). Overall, our results suggest that if, as predicted, low soil moisture events become more common in the Luquillo Mountains (Ramseyer et al., 2019), seedlings with more conservative resource‐acquisition strategies may be favored through differential rates of survival and growth. Nonetheless, complex responses are likely across vital rates and other sources of environmental heterogeneity (including disturbances) and will remain critically important for determining which species recruit into which microsites based on interactions between nutrient and light availability and soil moisture.

### 4.1 Correlations between demographic metrics

Interestingly, and contrary to our expectations, we found no evidence for associations between demographic metrics (sensitivity and tolerance) within or between vital rates, except for an expected relationship between growth sensitivity and tolerance that corresponds to a significant slope‐intercept correlation for linear models. This result highlights the complexity of demographic responses to variation in soil moisture; among species, responses, and thresholds to variation in soil moisture may vary across vital rates (Hogan et al., 2022; Uriarte et al., 2018). More work is required to untangle how complex demographic responses ultimately scale up to influence population trajectories in diverse communities.

### 4.2 Trait‐mediated demographic response to soil moisture gradient

A major approach of trait‐based ecology has focused on using plant traits to explain demographic responses to environmental heterogeneity, including drought (Comita & Engelbrecht, 2014; Harrison & LaForgia, 2019; Hiromi et al., 2012; Markesteijn, Poorter, Bongers, et al., 2011; Poorter & Markesteijn, 2008). Prior work has provided some general insight but few studies have linked trait variation with demographic responses along continuous gradients of soil moisture (but see Engelbrecht & Kursar, 2003). Additionally, potential trade‐offs in resource use strategies among species can result in a variety of constraints when responding to drought but few studies have considered multiple metrics when assessing demographic responses to drought (Russo et al., 2021).

We separately assessed how traits are related to the ability of species to tolerate low soil moisture and the magnitude of responses of species across the soil moisture gradient (i.e., sensitivity). The multivariate trait axis (RC1) corresponded to an acquisitive/conservative trait spectrum; higher values of RC1 were associated with more conservative strategies (e.g., higher carbon investment in leaf and root tissue; de la Riva et al., 2016; Eldhuset et al., 2013; Köcher et al., 2012). As hypothesized, we found a positive relationship between species‐mean position along RC1 and species average survival as well as survival tolerance to low soil moisture, indicating that species with more conservative traits had higher survival tolerance to drought, which is generally consistent with prior work (de la Riva et al., 2016; Niinemets, 2001; Tng et al., 2018). In contrast, the sensitivity of survival along the soil moisture gradient was not significantly related to either RC axis, suggesting that the consequences of low soil moisture for differential survival across species may be more likely to manifest at certain thresholds as opposed to gradual differences in responses across a soil moisture gradient.

The second multivariate trait axis (RC2) reflected investment in below‐ground tissue, with higher values associated with longer root systems, more root tips, and higher root‐to‐shoot dry mass ratio. Species with higher values of RC2 were less tolerant to low soil moisture in terms of growth, indicating that species with more extensive root systems had lower growth rates in drier soils. This finding is somewhat surprising in that, typically, plants with more extensive root systems have increased water absorption and nutrient uptake, as well as improved access to deeper soil layers, and thus may be particularly important for seedlings to survive during periods of low soil moisture. One possible explanation for our findings is that seedlings with larger root systems require more resources to maintain their root system, which comes at a relatively high expense of above‐ground growth. Notably, species with higher values of RC2 were also more sensitive to variation in soil moisture in terms of growth, suggesting that seedlings with more extensive roots could more efficiently capitalize on higher levels of soil moisture than those with smaller root systems. Although average growth rates were positively associated with total rooting depth, we found no significant relationships between RC1 (the acquisitive–conservative axis) and either growth tolerance or sensitivity. In other words, the acquisitive–conservative resource axis captured by the traits we measured did reflect species‐level differences in average growth but not variation in growth in response to the soil moisture gradient.

In this study, we focused on trait‐mediated responses to a soil moisture gradient for seedlings because they can be particularly sensitive to drought due to limited root systems and also because of the logistic challenges of conducting drought experiments with larger plants. Although there is substantial seasonal variation in rainfall in the Luquillo mountains, the forest is considered aseasonal when compared to other sites, thus trees occurring in this area may be expected to be more susceptible to drought effects compared to trees occurring in more seasonal forests. Nonetheless, analysis of the relationship between adult tree species variation in drought susceptibility and climate does not show this pattern (Smith‐Martin et al., 2023), and understanding the overall effects of drought on tropical forest communities will require greater integration with data from different life stages. For example, Visser et al. (2016) reported ontogenetic shifts in the relationships between functional traits and vital rates for tropical trees suggesting that different traits may be more or less important at different life stages. While we lack data on hydraulic vulnerability of adult trees for most of the species in our experiment, our results are generally consistent with recent work on adult trees of other species in the LEF (Smith‐Martin et al., 2022a, 2022b), which showed relationships between drought vulnerability and tree species with different successional associations. Specifically, early successional species—which typically have relatively acquisitive traits—were less drought tolerant than late successional species (which typically have relatively conservative traits). Our seedling results combined with recent data from later life cycle stages will hopefully prove useful in parameterizing ecosystem demographic models, which previously suggested that increasing drought could cause the LEF to transition from a carbon sink to a carbon source (Feng et al., 2018).

The timing of our study relative to recent hurricane disturbance also needs to be noted. Specifically, our study began just over 1 year after Hurricane María, the strongest storm to affect the area in 89 years (Uriarte et al., 2019). Previous studies have shown the importance of temporal and spatial variation in light availability in the understory in determining seedling fate (Comita & Hubbell, 2009; Uriarte et al., 2018). For example, Comita et al. (2010) documented how light availability varied after Hurricane Georges (1998) and how this temporal variation influenced overall seedling survival and the way in which it interacted, at times, with biotic factors such as the density of con‐ and heterospecific seedlings to determine survival. In our study, understory light levels were somewhat higher than typical for closed‐canopy forests. By elevating understory temperatures, high light availability could exacerbate negative impacts of soil drought on seedling demography via a combination of more soil evaporation and increased water demands from elevated photosynthesis (Hogan et al., 2022). Overall, disturbance history provides important context for interpreting the results of the experiment and for making predictions about potential responses to compound disturbances in the future (Zimmerman et al., 2021).

## 5 CONCLUSIONS

Our results suggest that more frequent and intense droughts in the Luquillo Mountains of Puerto Rico are likely to favor species and individuals within the tree seedling community with more conservative functional strategies. Ultimately, these changes have the potential to alter ecosystem processes (e.g., carbon cycling; Feng et al., 2018) as well as responses to future disturbances (Smith‐Martin et al., 2022a). Developing a further understanding of the implications of our results will require considering the effects of variability in soil moisture at other life stages and other disturbances, which may have synergistic impacts with drought.

## AUTHOR CONTRIBUTIONS

**David Matlaga:** Conceptualization (equal); data curation (equal); formal analysis (equal); funding acquisition (equal); supervision (equal); writing – original draft (equal); writing – review and editing (equal). **Roel Lammerant:** Conceptualization (equal); data curation (equal); formal analysis (equal); writing – original draft (equal); writing – review and editing (equal). **J. Aaron Hogan:** Data curation (equal); formal analysis (equal); writing – original draft (equal); writing – review and editing (equal). **María Uriarte:** Conceptualization (equal); writing – original draft (equal); writing – review and editing (equal). **Celimar Rodriguez Valle:** Data curation (equal); writing – original draft (equal); writing – review and editing (equal). **Jess K. Zimmerman:** Conceptualization (equal); data curation (equal); funding acquisition (equal); writing – original draft (equal); writing – review and editing (equal). **Robert Muscarella:** Conceptualization (equal); data curation (equal); formal analysis (equal); funding acquisition (equal); supervision (equal); writing – original draft (equal); writing – review and editing (equal).

## CONFLICT OF INTEREST STATEMENT

The corresponding author confirms on behalf of all authors that there have been no involvements that might raise the question of bias in the work reported or in the conclusions, implications, or opinions stated.

## Supporting information


Data S1

## Data Availability

All data associated with this manuscript are available on Github at https://github.com/bobmuscarella/Luquillo_LTER_Seedling_Drought_Experiment.
