# Correction to “Survival, growth, and functional traits of tropical wet forest tree seedlings across an experimental soil moisture gradient in Puerto Rico”

**DOI:** 10.1002/ece3.11190

**Published:** 2024-04-05

Matlaga, D., Lammerant, R., Hogan, J. A., Uriarte, M., Rodriguez‐Valle, C., Zimmerman, J. K., & Muscarella, R. (2024). Survival, growth, and functional traits of tropical wet forest tree seedlings across an experimental soil moisture gradient in Puerto Rico. *Ecology and Evolution*, 14, e11095. https://doi.org/10.1002/ece3.11095

In the acknowledgements section, the sentence that reads “*Support was also provided by the University of Puerto Rico and the International Institute for Tropical Forestry*.” incorrectly identified supporting organizations. This should have read: “*The USDA Forest Service International Institute of Tropical Forestry and University of Puerto Rico‐Río Piedras provided additional support*.”

We apologize for this error.

