# Supplementary material for: Survival, growth, and functional traits of tropical wet forest tree seedlings across an experimental soil moisture gradient in Puerto Rico
Source: Ecol Evol. 2024 Mar 3;14(3):e11095. doi: 10.1002/ece3.11095 (PMC10950389; doi:10.1002/ece3.11095)
Supplement: Supplementary file 1 — Data S1 [file ECE3-14-e11095-s001.docx]

**Survival, growth, and functional traits of tropical wet forest tree seedlings across an experimental soil moisture gradient in Puerto Rico**

David Matlaga^1*^, Roel Lammerant^2,3*^, J. Aaron Hogan^4^, María Uriarte^5^, Celimar Rodriguez Valle^6^, Jess K. Zimmerman^6^, Robert Muscarella^2†^

^1^ Susquehanna University, Department of Biology, 514 University Ave, Selinsgrove, PA 17870 USA

^2^ Uppsala University, Plant Ecology and Evolution, Norbyvägen 16, 752 36 Uppsala, Sweden

^3^ Tvärminne Zoological Station, University of Helsinki, Hanko, Finland

^4^ Department Biology, University of Florida, Gainesville FL, 32611 USA

^5^ Department of Ecology, Evolution and Environmental Biology, Columbia University New York, NY 10027 USA

^6^ Department of Environmental Sciences, University of Puerto Rico-Rio Piedras San Juan, PR 00975 USA

* Co-first authors

^†^ Corresponding author: robert.muscarella@ebc.uu.se

**Contents**

- **Tables S1-S10**
- **Figures S1-S5**
- **Methods supplement**

**Table S1 (Supplementary materials)**. Hypotheses relating to tropical tree seedling’s response to drought.

| **Trait** | **Units** | **Predicted response to low soil moisture (i.e., more conservative traits)** | **Rationale** | **References** |
| --- | --- | --- | --- | --- |
| Leaf mass per area (LMA) | g/m^2^ | **+** | High LMA values relate to high leaf tissue density which tends to increase tolerance towards water limitations. | (de la Riva et al., 2016; Niinemets, 2001) |
| Average leaf area | cm^2^ | **–** | The leaf boundary layer is thinner for smaller leaves, which lowers the risk of heat damage under high air temperatures and low soil water potential. | (Maréchaux et al., 2020; Wright et al., 2017) |
| Leaf thickness | mm | **?** | Leaf thickness is positively correlated with irradiance and shows a mixed response to drought. | (de la Riva et al., 2016; Niinemets, 2001; Tng et al., 2018) |
| Stem wood density (WD) | g/cm^3^ | **+** | Dense wood tends to be correlated with a lower risk of drought induced cavitation. | (Castro-Díez et al., 1998; Hacke et al., 2001; Markesteijn et al., 2011) |
| Leaf dry matter content (LDMC) | % | **+** | High LDMC values imply a low amount of water in leaf fresh mass and tend to relate with thicker and more rigid cell walls. | (Markesteijn et al., 2011) |
| Stem dry matter content (SDMC) | % | **+** | High SDMC values imply a low amount of water in stem fresh mass and tend to be correlated with a lower risk of drought induced cavitation. | (Castro-Díez et al., 1998; Hacke et al., 2001) |
| Root dry mass fraction | % | **+** | Under increasing drought stress, plants tend to allocate proportionately more biomass towards their root system. The increase in root mass fraction positively influences the ability to retrieve water from the soil. | (Eziz et al., 2017; Larson & Funk, 2016) |
| Specific root length (SRL) | cm/g | **–** | Low SRL values imply a higher investment in root tissue per length which tend to relate with a longer root lifespan and a possible lower risk of drought induced cavitation. | (Eissenstat & Yanai, 1997; Larson & Funk, 2016; McCormack et al., 2012; Pérez-Harguindeguy et al., 2013) |
| Root tissue density | g/cm^3^ | **+** | Dense root tissue implies narrow vessels with thick cell walls and a lower risk of drought induced cavitation. | (Eldhuset et al., 2013; Köcher et al., 2012) |
| Average root diameter | mm | **+** | High root diameter values imply a higher investment in root tissue per length which tend to relate with a longer root lifespan. | (McCormack et al., 2012; Wang et al., 2018) |
| Root length | Cm | **–** | Reduced transpiration and respiration rates under increasing soil moisture stress tend to associate with a reduction in carbon transportation from the leaves to the root system, which implies a decrease in root length. | (Brunner et al., 2015; Eldhuset et al., 2013; Ruehr et al., 2009) |
| Number of root tips | # | **–** | Reduced transpiration and respiration rates under increasing soil moisture stress tend to associate with a reduction in carbon transportation from the leaves to the root system, which implies a decrease in number of root tips. | (Brunner et al., 2015; Eldhuset et al., 2013; Ruehr et al., 2009) |
| Root depth | cm | **–** | Reduced transpiration and respiration rates under increasing soil moisture stress tend to associate with a reduction in carbon transportation from the leaves to the root system, which implies a decrease in root depth. | (Brunner et al., 2015; Eldhuset et al., 2013; Ruehr et al., 2009) |

**Table S2 (Supplementary materials)**. Successional status, peak timing of their germination (Uriarte et al. 2005, Comita et al. 2010, Taylor 1993) and authority for the eight study species.

| **Species**  **(Family)** | **Successional status** | **Peak germination** | **Authority** |
| --- | --- | --- | --- |
| *Cecropia schreberiana* (Urticaceae) | Pioneer | Late March | Miq. |
| *Schefflera morototoni* (Araliaceae) | Pioneer | Late February/early March | (Aubl.) Maguire, Steyerm. & Frondin |
| *Urera baccifera* (Urticaceae) | Pioneer | Late March | (L.) Guadich. Ex Wedd. |
| *Manilkara bidentata* (Sapotaceae) | Late | Mid-March | (A.DC.) A.Chev. |
| *Guarea guidonia* (Meliaceae) | Late | Late February | (L.) Sleumer |
| *Tetragastris balsamifera* (Burseraceae) | Late | Late February | Sw. Oken |
| *Inga laurina* (Leguminosae) | Secondary | Early January | Sw. Wild |
| *Prestoea acuminata* var*. montana* (Arecaceae) | Secondary | Late February/early March | (Graham) A.J.Hend. & Galeano |

**Table S3 (Supplementary materials).** Relationship between leaf length and leaf area for shadehouse grown seedlings.

| Species | Relationship | R^2^ | Leaves (n) | Seedlings (n) |
| --- | --- | --- | --- | --- |
| *Prestoea acuminata* var*. montana* | *y = 1.3478x - 4.3908*  (*F_1,19_ = 37.3, p<0.0001)* | 0.66 | 20 | 20 |
| *Inga Laurina* | y= 3.42x -10.296  (*F_1,236_ = 981.3, p<0.0001)* | 0.81 | 238 | 30 |
| *Tetragastris balsamifera* | y= 4.34x-17.536  (*F_1,82_ = 336.2, p<0.0001)* | 0.81 | 84 | 30 |
| *Guarea guidonia* | y= 2.821x-4.305  (*F_1,110_ = 703.7, p<0.0001)* | 0.87 | 112 | 30 |
| *Manilkara bidentata* | y= 3.4113x-6.06  (*F_1,66_ = 107.6, p<0.0001)* | 0.62 | 67 | 29 |
| *Urera baccifera* | y= 2.305x-3.05  (*F_1,79_ = 1181.9, p<0.0001)* | 0.98 | 80 | 16 |
| *Schefflera morototoni* | y= 2.377x-2.765  (*F_1,102_ = 1081.3, p<0.0001)* | 0.92 | 103 | 30 |
| *Cecropia schreberiana* | y= 2.393x-3.0614  (*F_1,83_ = 495.5, p<0.0001)* | 0.86 | 84 | 27 |

**Table S4 (Supplementary materials).** Correlation between intrinsic water-use efficiency (*iWUE*) and soil moisture for six of the eight study species. Due to low sample size at the conclusion of the experiment, resulting from high mortality, *Urera baccifera* and *Cecropia schreberiana* were not included in water-use efficiency measurements.

| **Species** | **Pearson´s *r*** | **p-value** |
| --- | --- | --- |
| *Inga laurina* | **-0.78** | **< 0.001** |
| *Tetragastris balsamifera* | **0.05** | **0.85** |
| *Manilkara bidentata* | **-0.61** | **< 0.05** |
| *Guarea guidonia* | **-0.60** | **< 0.05** |
| *Prestoea acuminata* var*. montana* | **-0.22** | **0.35** |
| *Schefflera morototoni* | **-0.71** | **< 0.001** |

**Table S5 (Supplementary materials).** Pairwise Pearson correlation of the 11 traits included in the principal component analysis (PCA) (leaf mass per area (LMA), leaf dry matter content (LDMC), specific root length (SRL), root tissue density (RTD). Correlations with a p < 0.05 indicated with bold and *.

|  | **Average leaf area** | **LMA** | **LDMC** | **Mean leaf thickness** | **Root dry mass fraction** | **Root length** | **Root Depth** | **Average root diam.** | **Root tips** | **SRL** | **RTD** |
| --- | --- | --- | --- | --- | --- | --- | --- | --- | --- | --- | --- |
| Average leaf area | 1 | **0.20*** | **0.35*** | 0,04 | -0.03 | **0.23*** | **0.17*** | 0.01 | **0.20*** | **-0.27*** | **0.32*** |
| LMA | **0.20*** | 1 | **0.67*** | **0.44*** | **-0.12*** | **0,38*** | **0.30*** | **-0.15*** | **0.38*** | **-0.39*** | **0.47*** |
| LDMC | **0.35*** | **0.67*** | 1 | **0,18*** | -0.07 | **0.43*** | **0.41*** | **-0.13*** | **0.41*** | **-0.47*** | **0.63*** |
| Mean leaf thickness | 0.04 | **0.44*** | **0.18*** | 1 | **-0.19*** | -0.01 | -0.01 | -0.01 | 0.01 | **-0.19*** | **0.09*** |
| Root dry mass fraction | -0.03 | **-0.12*** | -0.07 | -**0.19*** | 1 | **0.27*** | **0.30*** | **0.12*** | **0.25*** | **-0.17*** | **0.14*** |
| Root length | **0.23*** | **0.38*** | **0.43*** | -0.01 | **0.27*** | 1 | **0.70*** | **-0.36*** | **0.93*** | **0.17*** | **0.26*** |
| Root Depth | **0.17*** | **0.30*** | **0.41*** | -0.01 | **0.30*** | **0,70*** | 1 | **-0.16*** | **0.67*** | **-0.27*** | **0.31*** |
| Average root diam. | 0.01 | **-0.15*** | **-0.13*** | -0.01 | **0.12*** | **-0.36*** | **-0.16*** | 1 | **-0.38*** | **-0.31*** | **-0.18*** |
| Root tips | **0.20*** | **0.38*** | **0.41*** | 0.01 | **0.25*** | **0.93*** | **0.67*** | **-0.38*** | 1 | **-0.13*** | **0.25*** |
| SRL | **-0.27*** | **-0.39*** | **-0.47*** | **-0.19*** | **-0.17*** | **0.17*** | **-0.27*** | **-0.31*** | **-0.13*** | 1 | **-0.47*** |
| RTD | **0.32*** | **0.47*** | **0.63*** | **0.09*** | **0.14*** | **0.26*** | **0.31*** | **-0.18*** | **0.25*** | **-0.47*** | 1 |

**Table S6 (Supplementary materials).** Correlation of traits of eight seedling species in soil manipulation experiment to varimax rotated PCA dimensions.

| **Trait (units)** |  | **Abbreviation** | **RC1** | **RC2** |
| --- | --- | --- | --- | --- |
| Average leaf area (cm^2^) |  | AvgLeafArea | 0.47 | 0.09 |
| Leaf mass per area (g/m^2^) |  | LMA | 0.80 | 0.15 |
| Leaf dry matter content (%) |  | LDMC | 0.82 | 0.25 |
| Leaf thickness (mm) |  | LeafThicknessMean | 0.48 | -0.24 |
| Root dry mass fraction (%) |  | RootDryMassFraction | -0.12 | 0.42 |
| Root length (cm) |  | RootLength | 0.28 | 0.90 |
| Root depth (cm) |  | RootDepth | 0.32 | 0.74 |
| Average root diameter (mm) |  | RootAvgDiam | 0.05 | -0.50 |
| Total amount of root tips |  | RootTips | 0.27 | 0.89 |
| Specific root length (cm/g) |  | SRL | -0.72 | 0.05 |
| Root tissue density (g/cm^3^) |  | RTD | 0.71 | 0.19 |

**Table S7 (Supplementary materials).** Summary of Cox proportional hazard models for survival. Model structure and description in methods. Values given for the overall model determined by ‘penalized’ loglikelihood. HR is the hazard ratio.

| Species | Model component | Variable | X^2^ | p | AIC | Coef | HR | p | Std Dev |
| --- | --- | --- | --- | --- | --- | --- | --- | --- | --- |
| *Cecropia schreberiana* | Overall model |  | 51.95 | <0.0001 | 21.31 |  |  |  |  |
|  | Fixed effects | Soil moisture |  |  |  | -0.01 | 0.99 | 0.590 |  |
|  |  | Canopy openness |  |  |  | -0.02 | 0.97 | 0.520 |  |
|  |  | Starting LA |  |  |  | -0.04 | 0.96 | 0.470 |  |
|  | Random effects | Plot (intercept) |  |  |  |  |  |  | 0.413 |
| *Guarea guidonia* | Overall model |  | 82.68 | <0.0001 | 38.05 |  |  |  |  |
|  | Fixed effects | Soil moisture |  |  |  | -0.16 | 0.84 | <0.0001 |  |
|  |  | Canopy openness |  |  |  | 0.05 | 1.05 | <0.0001 |  |
|  |  | Starting LA |  |  |  | 0.00 | 1.00 | <0.0001 |  |
|  | Random effects | Plot (intercept) |  |  |  |  |  |  | 1.32 |
| *Inga laurina* | Overall model |  | 18.69 | <0.0001 | 12.69 |  |  |  |  |
|  | Fixed effects | Soil moisture |  |  |  | -0.36 | 0.69 | 0.0170 |  |
|  |  | Canopy openness |  |  |  | 0.41 | 1.52 | 0.0320 |  |
|  |  | Starting LA |  |  |  | -0.05 | 0.94 | 0.1800 |  |
|  | Random effects | Plot (intercept) |  |  |  |  |  |  | 0.01 |
| *Manilkara bidentata* | Overall model |  | 27.62 | <0.0001 | 12.35 |  |  |  |  |
|  | Fixed effects | Soil moisture |  |  |  | -0.21 | 0.81 | 0.0057 |  |
|  |  | Canopy openness |  |  |  | 0.02 | 1.03 | 0.8400 |  |
|  |  | Starting LA |  |  |  | -0.22 | 0.79 | 0.0780 |  |
|  | Random effects | Plot (intercept) |  |  |  |  |  |  | 0.96 |
| *Prestoea acuminata* var*. montana* | Overall model |  | 88.78 | <0.0001 | 44.70 |  |  |  |  |
|  | Fixed effects | Soil moisture |  |  |  | -0.14 | 0.86 | <0.0001 |  |
|  |  | Canopy openness |  |  |  | 0.00 | 1.00 | <0.0001 |  |
|  |  | Starting LA |  |  |  | -0.02 | 0.97 | <0.0001 |  |
|  | Random effects | Plot (intercept) |  |  |  |  |  |  | 1.06 |
| *Tetragastris balsamifera* | Overall model |  | 31.67 | 0.004 | 3.49 |  |  |  |  |
|  | Fixed effects | Soil moisture |  |  |  | -0.04 | 0.96 | 0.130 |  |
|  |  | Canopy openness |  |  |  | 0.04 | 1.04 | 0.470 |  |
|  |  | Starting LA |  |  |  | -0.01 | 0.98 | 0.230 |  |
|  | Random effects | Plot (intercept) |  |  |  |  |  |  | 0.75 |
| *Urera baccifera* | Overall model |  | 51.95 | <0.0001 | 21.31 |  |  |  |  |
|  | Fixed effects | Soil moisture |  |  |  | -0.03 | 0.97 | 0.0570 |  |
|  |  | Canopy openness |  |  |  | 0.09 | 1.10 | 0.0020 |  |
|  |  | Starting LA |  |  |  | 0.17 | 0.83 | 0.0048 |  |
|  | Random effects | Plot (intercept) |  |  |  |  |  |  | 0.41 |

**Table S8 (Supplementary materials).** Summary of lme growth model. R^2^C is the conditional R2 and is interpreted as a variance explained by the entire model, including both fixed and random effects.

| Species | Model component | Variable | R^2^C | AIC | Estimate | t-value | Variance |
| --- | --- | --- | --- | --- | --- | --- | --- |
| *Cecropia schreberiana* | Overall model |  | 0.385 | -103.133 |  |  |  |
|  | Fixed effects | Intercept |  |  | 0.160 | 2.195 |  |
|  |  | Soil moisture |  |  | 0.000 | 0.110 |  |
|  |  | Canopy openness |  |  | -0.003 | -0.887 |  |
|  |  | Starting LA |  |  | -0.019 | -7.302 |  |
|  | Random effects | Individual |  |  |  |  | 0.000 |
|  |  | Plot |  |  |  |  | 0.004 |
|  |  | Residual |  |  |  |  | 0.022 |
| *Guarea guidonia* | Overall model |  | 0.539 | 756.164 |  |  |  |
|  | Fixed effects | Intercept |  |  | 0.353 | 2.626 |  |
|  |  | Soil moisture |  |  | 0.014 | 3.939 |  |
|  |  | Canopy openness |  |  | 0.002 | 0.254 |  |
|  |  | Starting LA |  |  | -0.016 | -17.673 |  |
|  | Random effects | Individual |  |  |  |  | 0.037 |
|  |  | Plot |  |  |  |  | 0.019 |
|  |  | Residual |  |  |  |  | 0.109 |
| *Inga laurina* | Overall model |  | 0.547 | 756.164 |  |  |  |
|  | Fixed effects | Intercept |  |  | 0.335 | 1.068 |  |
|  |  | Soil moisture |  |  | 0.042 | 5.106 |  |
|  |  | Canopy openness |  |  | 0.050 | 2.627 |  |
|  |  | Starting LA |  |  | -0.012 | -18.494 |  |
|  | Random effects | Individual |  |  |  |  | 0.131 |
|  |  | Plot |  |  |  |  | 0.159 |
|  |  | Residual |  |  |  |  | 0.548 |
| *Manilkara bidentata* | Overall model |  | 0.518 | -647.259 |  |  |  |
|  | Fixed effects | Intercept |  |  | 0.230 | 4.005 |  |
|  |  | Soil moisture |  |  | 0.005 | 3.681 |  |
|  |  | Canopy openness Canopy openness |  |  | 0.006 | 1.759 |  |
|  |  | Starting LA |  |  | -0.012 | -19.352 |  |
|  | Random effects | Individual |  |  |  |  | 0.006 |
|  |  | Plot |  |  |  |  | 0.003 |
|  |  | Residual |  |  |  |  | 0.022 |
| *Prestoea* *montana* | Overall model |  | 0.436 | -644.417 |  |  |  |
|  | Fixed effects | Intercept |  |  | 0.071 | 1.331 |  |
|  |  | Soil moisture |  |  | 0.003 | 2.309 |  |
|  |  | Canopy openness |  |  | 0.006 | 2.064 |  |
|  |  | Starting LA |  |  | -0.013 | -13.307 |  |
|  | Random effects | Individual |  |  |  |  | 0.003 |
|  |  | Plot |  |  |  |  | 0.003 |
|  |  | Residual |  |  |  |  | 0.019 |
| *Tetragastris balsamifera* | Overall model |  | 0.247 | 1308.641 |  |  |  |
|  | Fixed effects | Intercept |  |  | 0.377 | 2.484 |  |
|  |  | Soil moisture |  |  | 0.007 | 1.991 |  |
|  |  | Canopy openness |  |  | 0.007 | 0.816 |  |
|  |  | Starting LA |  |  | -0.006 | -8.591 |  |
|  | Random effects | Individual |  |  |  |  | 0.0269 |
|  |  | Plot |  |  |  |  | 0.0168 |
|  |  | Residual |  |  |  |  | 0.260 |
| *Urera baccifera* | Overall model |  | 0.154 | -93.727 |  |  |  |
|  | Fixed effects | Intercept |  |  | 0.377 | 2.484 |  |
|  |  | Soil moisture |  |  | 0.007 | 1.991 |  |
|  |  | Canopy openness |  |  | 0.007 | 0.816 |  |
|  |  | Starting LA |  |  | -0.007 | -8.591 |  |
|  | Random effects | Individual |  |  |  |  | 0.027 |
|  |  | Plot |  |  |  |  | 0.016 |
|  |  | Residual |  |  |  |  | 0.260 |

**Table S9 (Supplementary materials).** Estimated coefficients [and 95% credible intervals] for associations between and among demographic metrics for different vital rates. Bold and asterisks indicate estimates where 95% CIs do not overlap zero; comparisons that were not made are shown as NA.

| **Comparison** | **Intercept [95% CIs]** | **Slope [95% CIs]** |
| --- | --- | --- |
| Average growth – Average survival | 45.21 [15.14, 76.77] | 28.54 [-7.46, 64.20] |
| Growth sensitivity – Growth tolerance | 0.04 [0.00, 0.09] | **-0.39 [-0.74, -0.13]*** |
| Growth sensitivity – Survival sensitivity | 0.28 [-0.16, 0.71] | 0.10 [-1.76, 2.65] |
| Survival sensitivity – Survival tolerance | 0.50 [-0.07, 1.07] | -0.31 [-1.96, 1.28] |
| Survival tolerance – Growth tolerance | 0.41 [-1.34, 1.97] | -4.71 [-131.48, 154.21] |

**Table S10 (Supplementary materials).** Estimated coefficients [95% credible intervals] for trait associations with species-level demographic rates and demographic metrics of tolerance and sensitivity. Bold and asterisks indicate estimates where 95% CIs do not overlap zero.

| **Trait** | **Average Survival** | **Average Growth** | **Growth**  **Sensitivity** | **Growth**  **Tolerance** | **Survival**  **Sensitivity** | **Survival**  **Tolerance** |
| --- | --- | --- | --- | --- | --- | --- |
| Log leaf area | 30.72 [-15.86, 78.79] | 0.94 [-0.41, 2.19] | 0.55 [-0.02, 1.09] | -0.01 [-0.7, 0.69] | -0.09 [-0.22, 0.01] | 0.4 [-0.03, 0.91] |
| Log LMA | 54.14 [-24.98, 145.28] | 1.17 [-0.99, 3.26] | **1.08 [0.17, 1.97]*** | 0.2 [-0.86, 1.33] | -0.1 [-0.38, 0.14] | 0.52 [-0.43, 1.49] |
| Log Leaf thickness | 76.9 [-43.81, 277.83] | 1.09 [-3.88, 6.07] | 1.92 [-1.07, 4.57] | 0.52 [-2.09, 2.91] | -0.07 [-0.75, 0.58] | 0.52 [-2.12, 3.16] |
| LDMC | 162.28 [-45.64, 367.74] | 4.3 [-1.11, 9.76] | **2.95 [1.01, 4.91]*** | -0.41 [-3.34, 2.64] | -0.37 [-1.05, 0.19] | 1.67 [-0.31, 4.21] |
| Log R:S ratio | 73.21 [-41.56, 182.78] | 1.09 [-2.78, 4.79] | 0.42 [-1.66, 2.49] | 0.81 [-0.52, 2.18] | **-0.24 [-0.55, -0.03]*** | 0.79 [-0.38, 2.09] |
| Log SRL | -18.78 [-77.5, 16.41] | -0.55 [-2.15, 0.99] | -0.78 [-1.56, 0.07] | 0.07 [-0.75, 0.91] | 0.1 [-0.04, 0.27] | -0.38 [-1.11, 0.34] |
| Log RTD | **93.21 [12.08, 174.73]*** | 1.64 [-0.62, 3.83] | **1.12 [0.28, 1.89]*** | -0.2 [-1.33, 0.99] | -0.12 [-0.37, 0.08] | 0.6 [-0.28, 1.64] |
| Log Root length | 31.35 [-20.3, 85.84] | 1.16 [-0.16, 2.39] | 0.55 [-0.14, 1.22] | 0.14 [-0.54, 0.87] | -0.15 [-0.3, -0.02]* | **0.54 [0.19, 1.02]*** |
| Log Root Avg Diam | 98 [-267.63, 470.45] | -2.73 [-14.3, 8.13] | 0.07 [-6.59, 6.46] | 1.23 [-3.81, 6.13] | -0.44 [-1.23, 0.33] | -0.11 [-5.64, 5.07] |
| Log Root Depth | 46.8 [-45.75, 174.77] | **3.12 [0.16, 5.67]*** | 1.4 [-0.26, 2.83] | -0.07 [-1.64, 1.57] | **-0.42 [-0.86, -0.09]*** | **1.49 [0.6, 2.62]*** |
| Log Root Tips | 17.31 [-28.3, 86.76] | 1.43 [-0.11, 2.82] | 0.63 [-0.29, 1.47] | 0.11 [-0.75, 0.96] | -0.16 [-0.41, 0.03] | **0.7 [0.16, 1.39]*** |
| RC axis 1 | **25.26 [8.53, 43.86]*** | 0.34 [-0.26, 0.9] | **0.28 [0.09, 0.47]*** | -0.02 [-0.29, 0.24] | -0.02 [-0.08, 0.03] | 0.12 [-0.07, 0.35] |
| RC axis 2 | 28.87 [-5.41, 65.77] | 0.68 [-0.03, 1.44] | 0.24 [-0.23, 0.65] | 0.06 [-0.35, 0.46] | -0.07 [-0.16, 0]* | **0.3 [0.07, 0.6]*** |

**Figure S1**. The experimental seedling plot design. Seedlings were planted in 80x80 cm plots using a 4x4 grid arrangement with 10 cm spacing and 40 cm exterior buffer area (left, drawing not to scale). Sixteen seedlings (two seedlings each of eight species) were assigned to locations within the grid randomly. Rainout shelters were constructed above each seedling plot (right). The rainout shelters were approximately two feet off the ground (exact height varied depending on topography) and constructed from plastic materials (see methods).

**
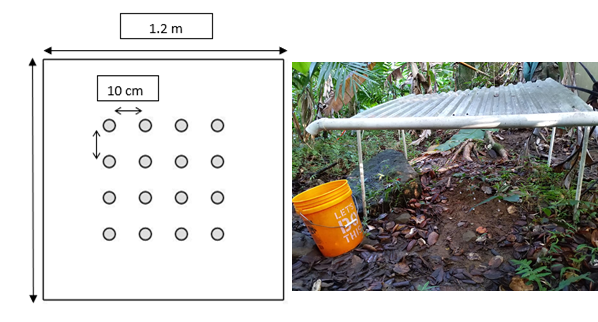
**

**Figure S2**. (A) Percent volumetric soil moisture (average of 4 measurements – one in each plot corner) for each seedling plot over time, recorded every two weeks (May 2019 - January 2020). Shelters where precipitation was excluded are colored red (i.e., the drought treatment, n = 30). Shelters where precipitation was collected and hand-watered back onto plots are colored blue (i.e., the control treatment, n =30). Panels (B) and (C) show the mean and minimum soil moisture recorded in each plot during the experiment by treatment.**
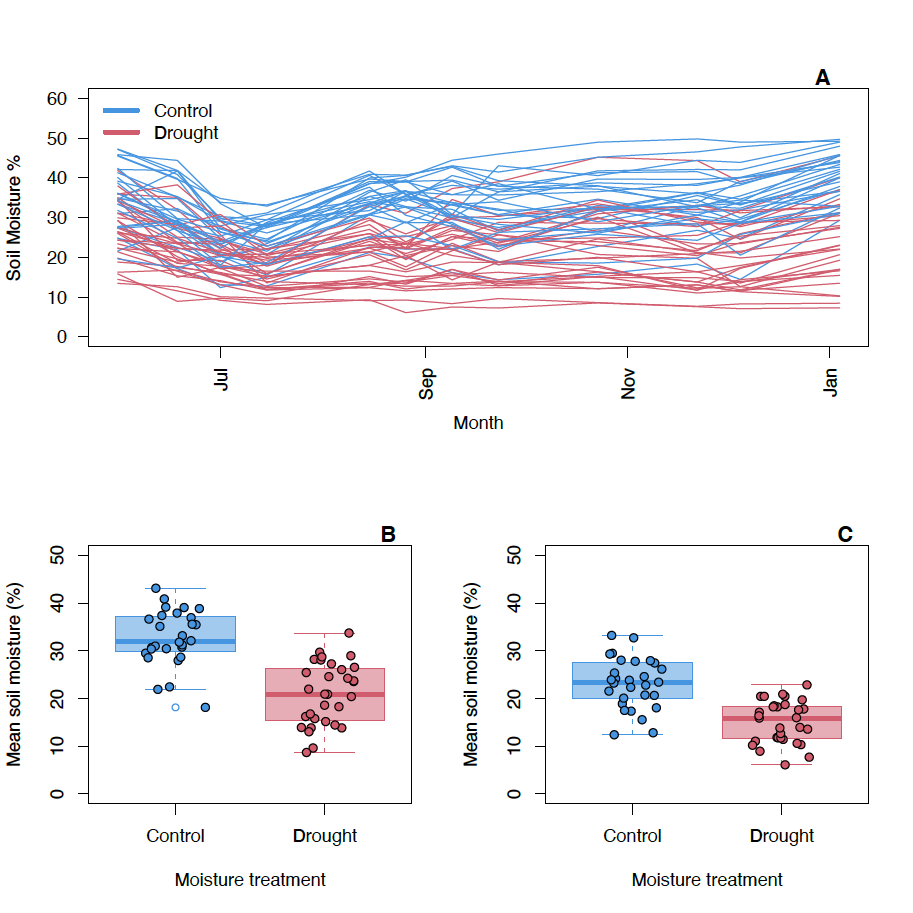
**

**Figure S3.** Relationships between intrinsic water-use efficiency (iWUE) of seedlings (N=120) and soil moisture for eight species. Solid lines show statistically-significance linear relationships for species (*Guarea guidonia*, *Inga laurina*, *Manilkara bidentata*, and *Schefflera morototoni*), whereas dotted lines show non-significant relationships (*Prestoea acuminata* var. *montana*, and *Tetragastris balsamifera*).


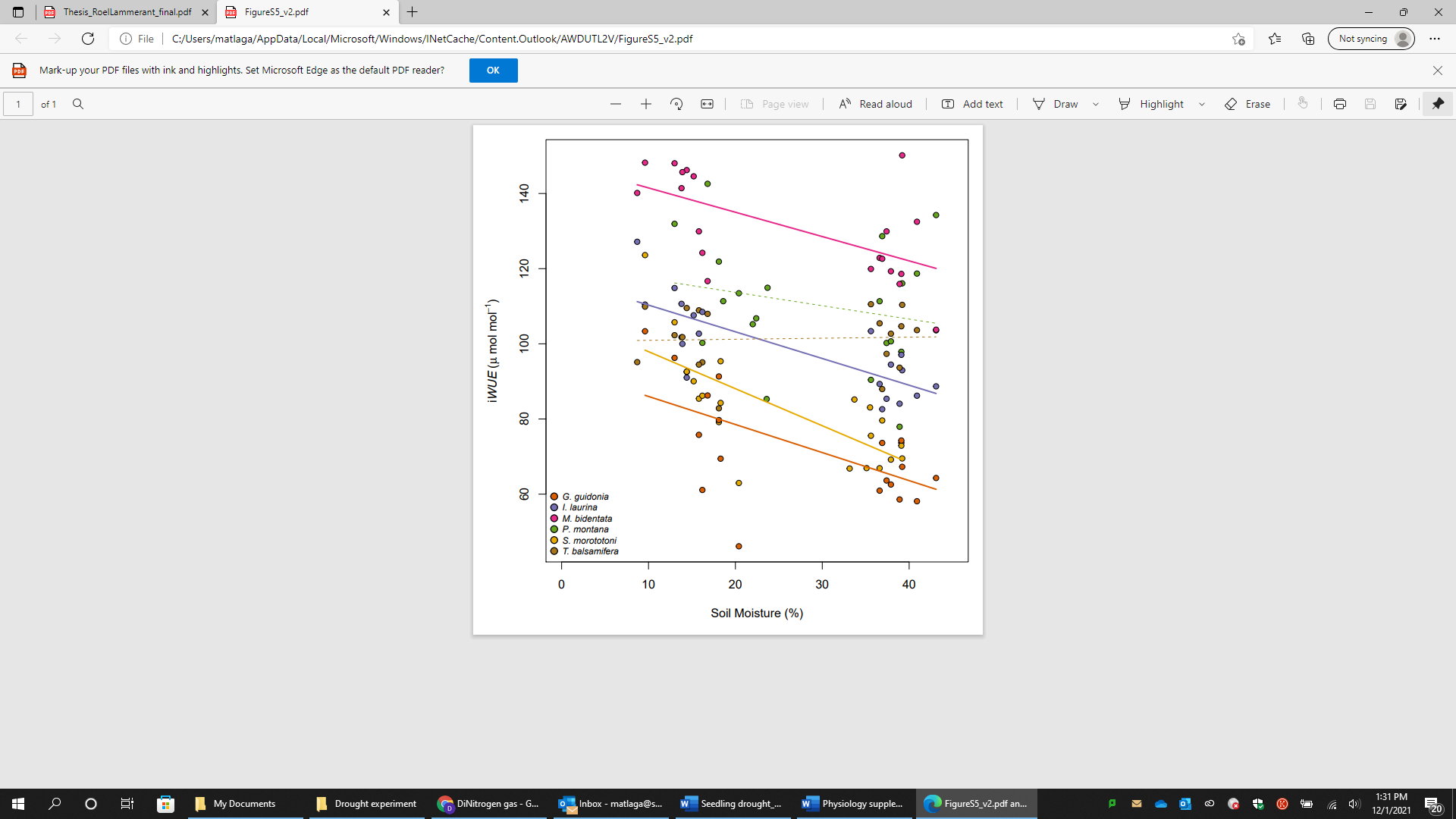


**Figure S4.** Survival for the eight species over the duration of the experiment.


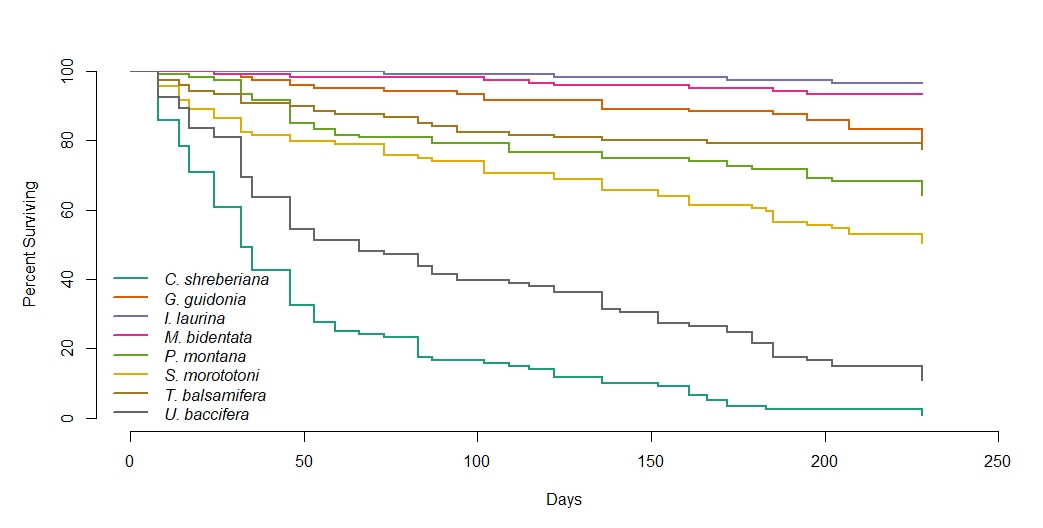


**Figure S5.** Total leaf area growth trajectories for all planted seedlings during the experiment. Lines are colored on a continuous scale indicating plot average soil moisture, with blue shades showing wetter plots and red shades showing drier plots.


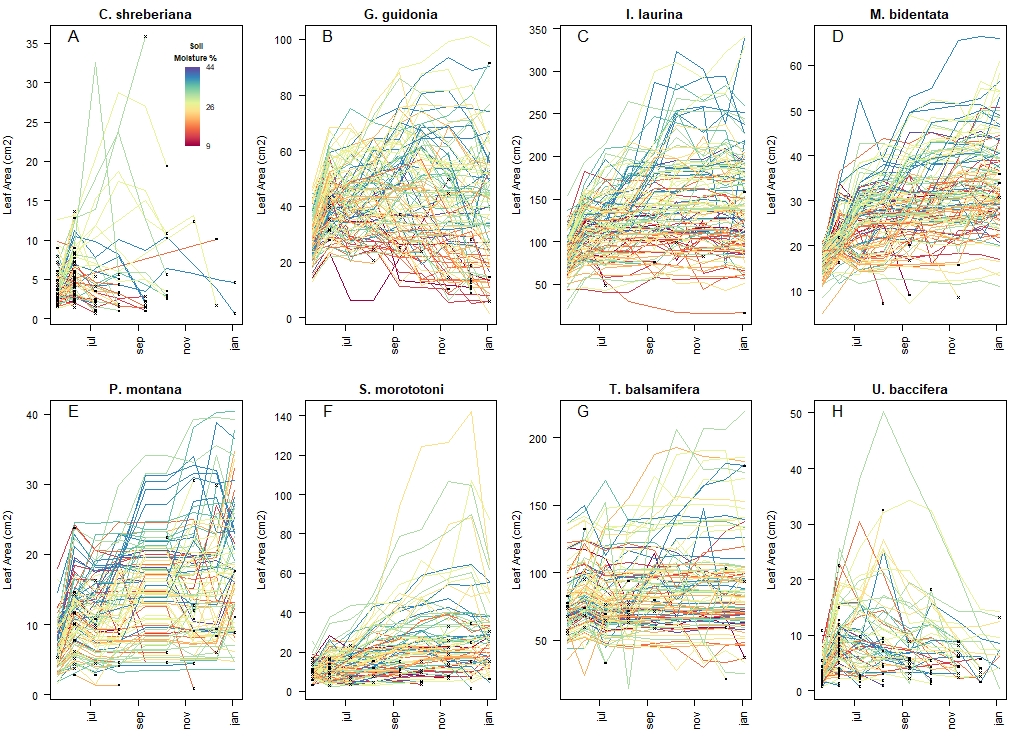


**Methods supplement (iWUE)**

To directly assess the effect of the experimental treatments on seedling physiological performance, we measured leaf intrinsic water-use efficiency (iWUE) using carbon (C) isotopes. Isotopically-derived iWUE is an indicator of plant carbon assimilation and *g_sw_* (stomatal conductance to water vapor slope) at intermediate time scales (weeks to months in the context of this experiment). It integrates drought stress through isotopically-derived estimates of intracellular CO_2_ (*c_i_*), which decreases as *g_sw_* decreases with increasing drought stress (Cernusak et al 2013 Farquhar et al. 1982). Leaf tissue elemental analyses of carbon (C), nitrogen (N) and their isotopes (^13^C and ^15^N) were completed on selected seedlings from the wettest and driest plots of the experimental soil moisture gradient. *U. baccifera* and *C. shreberiana* were not included in leaf tissue elemental analyses because of low overall survival. Ten individuals of each of the remaining six species were harvested from relatively wet and relatively dry plots (20, in total) and used for leaf elemental analysis. Dried leaf tissues were homogenized in individual 15 mL sterile plastic vials using sterile stainless-steel beads and a Mini-G 1600 tissue homogenizer (SPEX Sample Prep, Metuchen, NJ). Samples were analyzed using a Carlo Erba NA 1500 Elemental Analyzer (Thermo Scientific, Waltham, MA USA), fitted with a Costech zero-blank autosampler at the Duke Environmental Stable Isotope Laboratory (Durham, North Carolina).

Because the photosynthetic enzyme, Rubisco, fractionates ambient CO_2_ during carboxation at 4.4 ‰ (Farquhar et al. 1982), calculations, leaf δ^13^C can be used to calculate intrinsic *c_i_*. Lower intrinsic *c_i_* values typically indicate lower gsw, assuming a constant leaf mesophyll conductance and linear relationship of *c_i_* to *c_c_* (mole fraction of CO_2_ in the chloroplast). Carbon isotope values were converted to intrinsic (i.e., long-term) concentration of intracellular CO_2_ (*c_i_*) estimates using the following equation for C3 plants and a *c_a_* value of 408 μmols mol^-1^ CO_2_.: Δ= (4.4+2.26*c*_i_/*c_a_*) (Farquhar et al. 1982, Lambers Chapin and Pons 2008). Intrinsic water use efficiency (iWUE) was determined from calculated intrinsic c_i_ values using the following equation, from Lambers, Chapin and Pons (2008): iWUE = (c*_a_* *(1-*c*_i_/*c_a_*))/1.6, We used linear regressions to evaluate how seedling *iWUE* varied with soil moisture, fitting separate regressions for each of the six species for which were measured *iWUE*.

**References**

Brunner, I., Herzog, C., Dawes, M. A., Arend, M., & Sperisen, C. (2015). How tree roots respond to drought. *Frontiers in Plant Science*, *6*, 547.

Castro-Díez, P., Puyravaud, J. P., Cornelissen, J. H. C., & Villar-Salvador, P. (1998). Stem anatomy and relative growth rate in seedlings of a wide range of woody plant species and types. *Oecologia*, *116*(1), 57–66.

de la Riva, E. G., Olmo, M., Poorter, H., Ubera, J. L., & Villar, R. (2016). Leaf Mass per Area (LMA) and Its Relationship with Leaf Structure and Anatomy in 34 Mediterranean Woody Species along a Water Availability Gradient. *PloS One*, *11*(2), e0148788.

Eissenstat, D. M., & Yanai, R. D. (1997). The Ecology of Root Lifespan. In M. Begon & A. H. Fitter (Eds.), *Advances in Ecological Research* (Vol. 27, pp. 1–60). Academic Press.

Eldhuset, T. D., Nagy, N. E., Volařík, D., Børja, I., Gebauer, R., Yakovlev, I. A., & Krokene, P. (2013). Drought affects tracheid structure, dehydrin expression, and above- and belowground growth in 5-year-old Norway spruce. *Plant and Soil*, *366*(1), 305–320.

Eziz, A., Yan, Z., Tian, D., Han, W., Tang, Z., & Fang, J. (2017). Drought effect on plant biomass allocation: A meta-analysis. *Ecology and Evolution*, *7*(24), 11002–11010.

Hacke, U. G., Sperry, J. S., Pockerman, W. T., Davis, S. D., & McCulloh, K. A. (2001). Trends in wood density and structure are linked to prevention of xylem implosion by negative pressure. *Oecologia*, *126*, 457–461.

Köcher, P., Horna, V., Beckmeyer, I., & Leuschner, C. (2012). Hydraulic properties and embolism in small-diameter roots of five temperate broad-leaved tree species with contrasting drought tolerance. *Annals of Forest Science*, *69*(6), 693–703.

Larson, J. E., & Funk, J. L. (2016). Seedling root responses to soil moisture and the identification of a belowground trait spectrum across three growth forms. *The New Phytologist*, *210*(3), 827–838.

Maréchaux, I., Saint-André, L., Bartlett, M. K., Sack, L., & Chave, J. (2020). Leaf drought tolerance cannot be inferred from classic leaf traits in a tropical rainforest. *The Journal of Ecology*, *108*(3), 1030–1045.

Markesteijn, L., Poorter, L., Paz, H., Sack, L., & Bongers, F. (2011). Ecological differentiation in xylem cavitation resistance is associated with stem and leaf structural traits. *Plant, Cell & Environment*, *34*, 137–148.

McCormack, L., Adams, M., Thomas, S., Smithwick, E. A. H., & Eissenstat, D. M. (2012). Predicting fine root lifespan from plant functional traits in temperate trees. *The New Phytologist*, *195*(4), 823–831.

Niinemets, Ü. (2001). Global-scale climatic controls of leaf dry mass per area, density, and thickness in trees and shrubs. *Ecology*, *82*(2), 453–469.

Pérez-Harguindeguy, N., Díaz, S., Garnier, E., Lavorel, S., Poorter, H., Jaureguiberry, P., Bret-Harte, M. S., Cornwell, W. K., Craine, J. M., Gurvich, D. E., Urcelay, C., Veneklaas, E. J., Reich, P. B., Poorter, L., Wright, I. J., Ray, P., Enrico, L., Pausas, J. G., de Vos, A. C., … Cornelissen, J. H. C. (2013). New handbook for standardised measurement of plant functional traits worldwide. *Australian Journal of Botany*, *61*(3), 167–234.

Ruehr, N. K., Offermann, C. A., Gessler, A., Winkler, J. B., Ferrio, J. P., Buchmann, N., & Barnard, R. L. (2009). Drought effects on allocation of recent carbon: from beech leaves to soil CO2 efflux. *The New Phytologist*, *184*(4), 950–961.

Tng, D. Y. P., Apgaua, D. M. G., Ishida, Y. F., Mencuccini, M., Lloyd, J., Laurance, W. F., & Laurance, S. G. W. (2018). Rainforest trees respond to drought by modifying their hydraulic architecture. *Ecology and Evolution*, *8*(24), 12479–12491.

Wang, R., Wang, Q., Zhao, N., Xu, Z., Zhu, X., Jiao, C., Yu, G., & He, N. (2018). Different phylogenetic and environmental controls of first‐order root morphological and nutrient traits: Evidence of multidimensional root traits. *Functional Ecology*, *32*(1), 29–39.

Wright, I. J., Dong, N., Maire, V., Prentice, I. C., Westoby, M., Díaz, S., Gallagher, R. V., Jacobs, B. F., Kooyman, R., Law, E. A., Leishman, M. R., Niinemets, Ü., Reich, P. B., Sack, L., Villar, R., Wang, H., & Wilf, P. (2017). Global climatic drivers of leaf size. *Science*, *357*(6354), 917–921.
